# Role and Possible Mechanisms of Sirt1 in Depression

**DOI:** 10.1155/2018/8596903

**Published:** 2018-01-31

**Authors:** Guofang Lu, Jianguo Li, Hongmei Zhang, Xin Zhao, Liang-Jun Yan, Xiaorong Yang

**Affiliations:** ^1^National Key Disciplines, Key Laboratory for Cellular Physiology of Ministry of Education, Department of Neurobiology, Shanxi Medical University, No. 56 Xin Jian South Road, Taiyuan, Shanxi 030001, China; ^2^Department of Environmental Health, Shanxi Medical University, No. 56 Xin Jian South Road, Taiyuan, Shanxi 030001, China; ^3^Department of Pharmaceutical Sciences, UNT System College of Pharmacy, University of North Texas Health Science Center, Fort Worth, TX 76107, USA

## Abstract

Depression is a common, devastating illness. Due to complicated causes and limited treatments, depression is still a major problem that plagues the world. Silent information regulator 1 (Sirt1) is a deacetylase at the consumption of NAD^+^ and is involved in gene silencing, cell cycle, fat and glucose metabolism, cellular oxidative stress, and senescence. Sirt1 has now become a critical therapeutic target for a number of diseases. Recently, a genetic study has received considerable attention for depression and found that Sirt1 is a potential gene target. In this short review article, we attempt to present an up-to-date knowledge of depression and Sirt1 of the sirtuin family, describe the different effects of Sirt1 on depression, and further discuss possible mechanisms of Sirt1 including glial activation, neurogenesis, circadian control, and potential signaling molecules. Thus, it will open a new avenue for clinical treatment of depression.

## 1. Depression

Depression, also known as depressive disorder, is caused by a variety of factors, and its main symptoms are psychological or emotional disorders. Depression is a chronic and life-threatening mental disorder, affecting almost 350 million people worldwide and places a huge burden on both individuals and families [1, 2]. Moreover, depression could be lethal due to the possibility of suicide [3]. Depression can also increase risks of cardiac dysfunction, cerebrovascular disease, and other underlying mechanisms of mortality.

There are several ways of classification of depression. According to the severity of symptoms, depression can be divided into mild depression and major depressive disorder (MDD), or according to the timing of symptoms, it can be divided into acute depression and chronic depression. As the etiology of depression involves a variety of physiological and psychological factors, it is hard to treat this disease. Antidepressants are the primary means of treating depression, and amitriptyline and sertraline are mainly used for this disease. While it is known that antidepressant medications exhibit side effects and come with safety concerns, withdrawal of the medications could also be very difficult. Therefore, any use of medication requires close monitoring. Psychological therapy is also an effective treatment for clinical depression and may prevent a person with mild depression from becoming more severely depressed, but the effect is subject to many factors. In addition to antidepressants and psychological therapy, electroconvulsive therapy (ECT) and vagus nerve stimulation (VNS) are also new solutions for depression, although more efficient, they also have significant adverse effects including retrograde amnesia [4]. All of these depression treatments are thought to be effective; however, these treatments take time, and recovery usually has its ups and downs. Furthermore, the same treatment may have different effects for different patients.

In the past few decades, people have explored a variety of molecules, cells, and signaling pathways involved in depression, but the pathogenesis of depression still remains to be elucidated. Because of complicated causes and limited treatments, depression is still one of the scary diseases for humans. Indeed, numerous researchers have focused on the genetic study of depression and found some potential gene targets. Sirt1 is one of these genes.

## 2. Sirt1

Sirtuins are conserved proteins found in all aerobic organisms. They are nicotinamide-adenine dinucleotide- (NAD^+^-) dependent deacylases and originally recognized as class three histone deacetylases (HDAC) [5]. Silent information regulator 2 (Sirt2) is the first known sirtuin identified in *Saccharomyces cerevisiae* whereby it is shown to prolong the life span of yeast. In mammals, seven human Sirt2 homologues (sirtuins) designated as Sirt1 to Sirt7 have been identified to date. All of the seven sirtuin proteins share a conserved catalytic core. The central catalytic core is a 245 amino acid peptide. The core is a large domain that is typical for NAD^+^-dependent proteins and a small Zn^2+^ ribbon motif containing the consensus sequence Cys-X2–4Cys-X15–40-Cys-X2–4-Cys and an *α*-helical region. Sirtuins are associated with calorie restriction, aging, metabolism, cancer, transcriptional silencing, chromosomal stability, cell differentiation, stress response, inflammation, apoptosis, DNA repair, and prevention of age-related ocular diseases [6–11].

Among these sirtuins, Sirt1 is well studied due to its similarity to Sirt2 and its potential protective role in vascular disease. The deacetylase domain of Sirt1 is highly structured and while the N- and C-terminals are very flexible. This structural feature allows it to offer more modulation sites such as posttranslational modifications and interaction with ligands and proteins. The gene encoding Sirt1 is located at 10q21.3, and the length is 33,715 bp, with nine exons encoding 747 amino acids [12]. As an NAD^+^-dependent HDAC, Sirt1 can deacetylate numerous substrates such as transcription factors, histones, and many other enzymes [13]. Sirt1 is known to be involved in a number of physiological processes, including apoptosis, cell differentiation, development, autophagy, and cancer metabolism, as well as circadian rhythms [14–19]. Recently, it has been shown that Sirt1 plays a vital role in higher-order brain dysfunctions such as drug addiction [20], endocrine regulation [18, 21], and synaptic plasticity [22, 23]. Furthermore, there are pieces of evidence suggesting that Sirt1 plays a critical role in cardiovascular diseases [24], metabolic and health span [25, 26], neurodegenerative diseases [27, 28], cancer [29], and optic neuritis [30].

## 3. Role of Sirt1 in Depression

The CONVERGE (China, Oxford and Virginia Commonwealth University Experimental Research on Genetic Epidemiology) consortium recruited eleven thousand Han Chinese women through a collaboration involving nearly 60 hospitals in China. The study found two loci that contributed to the risk of MDD on chromosome 10: one is close to the Sirt1 gene (P52.53310210) and the other exists in one of the introns of the LHPP gene (P56.45310212) [31]. Additionally, a case-control investigation in Japan showed that one tagging SNP (rs10997875) in the Sirt1 gene could play a role in MDD pathophysiology [32]. It was also found that there is a link between the Sirt1 gene (rs3758391) and depressive disorders [33]. Further, it has been shown that Sirt1 expression in the peripheral blood from individuals with depression is significantly less than those in healthy subjects [32]. Similarly, Sirt1 expression is markedly downregulated in the blood of MDD patients when compared with control subjects and those with remitted MDD cases [34]. Based on the above summarized studies, we believe that Sirt1 plays an important role in depression.

Recent studies using animal models of depression also found that dysregulation of Sirt1 signaling has a critical role in depression-like behaviors [2]. Abe-Higuchi et al. reported that Sirt1 activity in the dentate gyrus is decreased upon chronic stress, and pharmacologic or genetic ablation of hippocampal Sirt1 resulted in an elevation in depression-like behaviors. When Sirt1 was activated, development of depression-related phenotypes and abnormal dendritic structures induced by chronic stress could be blocked [2]. In an animal model of depression, treatment with resveratrol that is a well-known Sirt1 activator can promote antidepressant effects in Wistar-Kyoto (WKY) rats [35]. In addition, the compound resveratrol was shown to attenuate depression-like behaviors in mice, which were triggered by repeated corticosterone [36] and lipopolysaccharide (LPS) [37]. It was also found that resveratrol can improve hyperanxiety status in high fructose-induced predisposing diabetic rats [38]. Resveratrol is best known for its ability to activate Sirt1. For example, Howitz et al. indicated that resveratrol can promote cell survival by stimulating Sirt1 in vivo [39]. And Hayashi et al. demonstrated that “treatment of porcine oocytes with resveratrol, which is an activator of Sirt1, upregulated the mitochondrial synthesis and degradation as well as improved the developmental abilities of the oocytes” [40]. In addition, Wang et al. showed that “resveratrol is an activator of Sirt1 (a member of the sirtuin family), which is an NAD^+^-dependent deacetylase that regulates various genes and protein involved in cell proliferation, apoptosis, senescence, and differentiation” [41]. Notwithstanding, it should be pointed out that resveratrol may be indirectly involved in Sirt1 activation. For example, it has been reported that resveratrol may also act indirectly through other signaling molecules [42].

Nonetheless, there are also conflicting reports showing the opposite effect of Sirt1. For example, it was demonstrated that mice with brain-specific Sirt1 knockout decreased anxiety and developed resilience to depression induced by social defeat, while mice with global Sirt1 overexpression had elevated anxiety and increased susceptibility to depression [43]. Likewise, Ferland and Schrader found that the protein levels and activities of Sirt1 in the hippocampal CA3 and DG regions were elevated following chronic stress in rats and the acetylation levels of the two regions were significantly decreased. The Sirt1 inhibitor sirtinol could restore histone acetylation, then improve the behavior of depression [44]. The reason for the discrepancy between Sirt1 protein expression and enzymatic function is unclear, but one possibility could be due to the utilized mice having different genetic backgrounds. Abe-Higuchi et al. used BALB/c mice, whereas Libert et al. used mixed genetic background mice back-crossed with C57BL/6 [43]. A second possibility is the different regional and cellular specificity of the manipulations carried out in the two studies. Third, Sirt1 could be heavily regulated by posttranslational modifications. Additionally, different stress protocols used between the studies could also lead to differences in Sirt1 expression [2].

## 4. Possible Mechanisms of Sirt1 in Depression

### 4.1. Sirt1 and Glial Activation

In the course of stress-induced depression, microglial activation is a key link, and depression is also considered as a microglia disease in many studies. It has been shown that brain microglial activation caused by nerve inflammation plays an important role in depression [45]. In LPS-induced depression model of mice, resveratrol attenuated LPS-dependent overactivation of microglia in the dentate gyrus-subgranular zone (DG-SGZ) [46]. Additionally, Kodali et al. reported that activated microglia and astrocyte hypertrophy were among the most obvious structural alterations in the hippocampus of old male F344 rats and these alterations contributed to age-associated memory loss and mood impairments. Resveratrol treatment reduced astrocyte hypertrophy and microglia activation in old rats [47]. Thus, activation of Sirt1 could improve mood function and play an antidepressant effect.

### 4.2. Sirt1 and Neurogenesis

It has been reported that dysfunctional hippocampal neurogenesis exists in certain rodent and nonhuman primate models of depression including chronic unpredictable mild stress, repeat restraint stress, social isolation, social defeat stress, and LPS treatment or administration of corticosterone [48–50]. In a recent study, Liu et al. demonstrated that resveratrol-triggered Sirt1 activation reverses LPS-induced depression-like behaviors by augmenting hippocampal neurogenesis [46]. Similarly, it has been shown that resveratrol treatment increased neurogenesis in the hippocampus of aged rats and prevented aging-associated memory loss and mood dysfunction [47]. These cases pointed out that resveratrol can ameliorate depression-like behaviors via protecting hippocampal neurogenesis. In addition, treatment with antidepressant drugs enhances hippocampal neurogenesis, and it has been suggested that increasing adult hippocampal neurogenesis could serve as a new drug target for the design of future antidepressant medications [51–53].

### 4.3. Sirt1 and Circadian Rhythm

Mammalian physiology and behavior are governed by an internal time-keeping system, the so-called circadian rhythm. Circadian rhythm occurs on the basis of the biological clock, which resides in the hypothalamic suprachiasmatic nucleus (SCN), with the regulation of metabolic, endocrine, and sleep rhythm. The positive elements of the circadian clock encompass two transcription factor family members, CLOCK and BMAL1 [54]. Sirt1 in the central nervous system has been shown to govern central circadian regulation by activating BMAL1 and CLOCK [55]. Asher et al. and Nakahata et al. demonstrated that Sirt1 can deacetylate and counteract the activity of the circadian clock [17, 56, 57]. Also, Sirt1, as a rheostat of circadian function, relays signals from cellular metabolites to the circadian clock. Additionally, it was evidenced that Sirt1 plays a role in circadian control in the model of liver-specific Sirt1 mutant mice in vivo [57].

Genomic studies have pinpointed circadian gene polymorphisms that influence the susceptibility of psychiatric disease [58, 59]. Moreover, a transcriptome-wide study of postmortem brains from MDD patients revealed weaker circadian gene expression and impaired phase relationships between individual clock genes [60]. Patients with MDD exhibit disrupted circadian rhythmicity in body temperature; hormone secretions, for example, cortisol and melatonin; blood pressure; and sleep-wake cycles [61, 62]. These suggest that depression is associated with circadian dysfunction. Recently, it has been found that Sirt1 protein expression and functional activity in a mouse model of depression have a shift in the rhythm due to chronic stress exposure [2]. These studies have shown that Sirt1 can affect circadian rhythm, which in turn contributes to depression.

### 4.4. Sirt1 and Signaling Molecules

Abe-Higuchi et al. investigated the function of extracellular signal-regulated protein kinases 1 and 2 (ERK1/2) as potential downstream targets of Sirt1 in a mouse model of depression induced by chronic stress. It was found that activation of hippocampal Sirt1 enhanced the ERK1/2 phosphorylation level under stressed condition and viral-modulated hippocampal ERK2 function contributed to antidepressive and prodepressive behaviors [2]. Another study revealed that chronic variable stress (CVS) increased depressive-like behavior and such changes accorded with an overall decrease in ERK1/2 phosphorylation, B cell lymphoma/leukemia-2 (Bcl-2) expression, and H4 (K12) acetylation in the hippocampal subregions upon chronic stress. However, there is no significant effects of sirtinol or stress on Akt phosphorylation [63]. Conversely, the effect of CVS on ERK1/2 phosphorylation in the amygdala has not been detected, but ERK1/2 phosphorylation is elevated in the medial prefrontal cortex and no changes in Bcl-2 levels could be detected. This indicates that activation of ERK1/2 and downstream pathways under stress condition are region-specific.

Finally, it is well known that brain-derived neurotrophic factor (BDNF) is involved in mental regulation and effectiveness of antidepressant medications [64–67]. It was reported that chronic ketamine administration in WKY rats also results in antidepressant effects, which is linked with an increased hippocampal BDNF expression [68]. In the same way, the antidepressant effect of resveratrol may be associated with hippocampal BDNF activation in an animal model of depression [35].

Also, it should be noted that Sirt1 and Sirt2 may play opposite roles in the brain. It has been reported that Sirt1 is mostly believed to be neuroprotective [69] while Sirt2 may enhance or facilitate neurodegeneration [70] in stress-related psychiatric disorders including depression. This opposite role of Sirt1 and Sirt2 is thought to be age-dependent, and the balance of these two Sirts might be crucial in part for the regulation of depression by Sirt1 [71, 72].

## 5. Summary

The rate of incidence, injury, and mortality of depression is high; however, drug therapy, psychotherapy, ECT, and VNS do not have a good therapeutic effect. This is largely due to the fact that the pathogenesis of depression has not been fully understood and the underlying biological mechanisms remain sketchy. Until now, depression is still a problem that plagues the world. Therefore, we need a comprehensive understanding of the cause of depression. In recent years, some studies have demonstrated that epigenetic regulation of depression plays a vital role in the pathogenesis of depression. HDACs are involved in the deacetylation of the gene nucleosomal histone, thereby affecting the epigenetic inheritance of the gene. Sirt1 belongs to the III HDACs that has been linked to various pathophysiological conditions, including depression. Sirt1, an NAD^+^-dependent deacetylase, has been extensively studied for its connection to depression, but the specific role of Sirt1 remains controversial. This review describes the different effects of Sirt1 on depression and possible mechanisms (Table 1) and points to the direction that Sirt1 could serve as a novel therapeutic target for clinical treatment of depression.

## Figures and Tables

**Table 1:** Sirt1 mechanisms involved in depression discussed in this paper.

Mechanisms	Effects	References
Glial activation	Depression	[41–43]
Neurogenesis	Depression	[42–49]
Circadian rhythm	Depression	[2, 17, 50–58]
Signaling molecule	Depression	[2, 35, 59–64]

## Abbreviations

Sirt1: Silent information regulator 1
MDD: Major depressive disorder
ECT: Electroconvulsive therapy
VNS: Vagus nerve stimulation
NAD^+^: Nicotinamide-adenine dinucleotide
HDAC: Histone deacetylases
Sirt2: Silent information regulator 2
LPS: Lipopolysaccharide
DG-SGZ: Gyrus-subgranular zone
SCN: Suprachiasmatic nucleus
ERK1/2: Extracellular signal-regulated protein kinases 1 and 2
CVS: Chronic variable stress
Bcl-2: B cell lymphoma/leukemia-2
BDNF: Brain-derived neurotrophic factor.
